# Mitochondrial DNA Haplogroup Confers Genetic Susceptibility to Nasopharyngeal Carcinoma in Chaoshanese from Guangdong, China

**DOI:** 10.1371/journal.pone.0087795

**Published:** 2014-01-31

**Authors:** Sheng-Ping Hu, Ju-Ping Du, De-Rui Li, Yong-Gang Yao

**Affiliations:** 1 Molecular Biology and Forensic Genetics Laboratory, Shantou University Medical College, Shantou, Guangdong, China; 2 Tumor Hospital, Shantou University Medical College, Shantou, Guangdon, China; 3 Key Laboratory of Animal Models and Human Disease Mechanisms of the Chinese Academy of Sciences and Yunnan Province, Kunming Institute of Zoology, Kunming, Yunnan, China; IPATIMUP (Institute of Molecular Pathology and Immunology of the University of Porto), Portugal

## Abstract

Recent studies have shown association of mtDNA background with cancer development. We analyzed mitochondrial DNA (mtDNA) control region variation of 201 patients with nasopharyngeal carcinoma (NPC) and of 201 normal controls from Chaoshan Han Chinese to discern mtDNA haplogroup effect on the disease onset. Binary logistic regression analysis with adjustment for gender and age revealed that the haplogroup R9 (*P* = 0.011, OR = 1.91, 95% CI = 1.16–3.16), particularly its sub-haplogroup F1 (*P* = 0.015, OR = 2.43, 95% CI = 1.18–5.00), were associated significantly with increased NPC risk. These haplogroups were further confirmed to confer high NPC risk in males and/or individuals ≥40 years of age, but not in females or in subjects <40 years old. Our results indicated that mtDNA background confers genetic susceptibility to NPC in Chaoshan Han Chinese, and R9, particularly its sub-haplogroup F1, is a risk factor for NPC.

## Introduction

Nasopharyngeal carcinoma (NPC) is an epithelial malignancy with a strikingly ethnic and geographic distribution [1]. It is also nicknamed “Canton cancer” since the world highest incidence is observed in Guangdong (Canton) Province in southern China. The incidence rate in southern China can be as high as 20 to 50 per 100,000 person-years, which is about 100 times higher than that in most other parts of the world [2]–[4]. Chaoshan (Teochew) is a littoral region located in the eastern part of Guangdong, and the people residing in this area are defined as Chaoshanese as they speak in a unique dialect and have a distinct lifestyle. The temporal age-standardized incidence rate of NPC in Chaoshan is 4.45/100,000 person-years from 1995 to 2004 [5], representing the second most common cancer in this population, and Chaoshan therefore can be classified as a medium-NPC-incidence area.

NPC is difficult to be detected in the early stage and radio-therapy treatment does not prevent metastasis and recurrence after treatment when the tumor is in the advanced stage [6], [7]. At present, tests for EBV-IgA-VCA and IgA titers to EBV capsid antigen have been widely used in clinical diagnoses of NPC. However, such tests fail to diagnose early NPC and are not useful for prognostic assessment [8], [9]. Therefore, it is important to explore new molecular markers for early diagnosis and prevention.

Mitochondria are essential organelles in eukaryotic cells that generate cellular energy through oxidative phosphorylation. Due to the lack of sophisticated DNA repair system and continual exposure to high levels of reactive oxygen species, mitochondrial DNA (mtDNA) is more susceptible to oxidative damage and harbors a greater number of mutations than nuclear DNA [10], [11]. It is proposed that cancer originates from a non-neoplastic cell which adopts anaerobic metabolism as a means of survival after injury to its respiratory system [12]. Thus mtDNA mutations that cause defects in mitochondrial respiratory enzyme complexes are thought to increase production of reactive oxygen species, which may contribute to cancer development and progression [10], [13], [14]. A number of studies have reported a positive association between mtDNA alterations (mutations, deletions, and instability) and various cancers [10], [14]–[16], and the observed mutational pattern on the cancerous mtDNAs might be best explained as relaxation of negative selection [17]. Since the copy number of mtDNA in a cell is much higher than that of the nuclear DNA, it is supposed to be significantly easier to analyze mtDNA than to analyze nuclear DNA. mtDNA, therefore, could be a good candidate acting as a potential useful biomarker to detect cancer-specific mutations.

The mtDNA haplogroup is defined by a group of mtDNAs that share a string of ancient polymorphisms and present continent-specific distributions. Such distribution specificity is also observed within China, in which the haplogroup pattern varies between southern and northern China, as well as among different ethnic populations [18]. Importantly, accumulating lines of evidence indicate that mtDNA haplogroups confer genetic susceptibility to human diseases, and various mtDNA haplogroups have been identified as risk/protective factors in a variety of cancers [19]–[22], including esophageal carcinoma in the Chaoshan population [21], [23]. With regard to NPC, studies reported thus far focus mainly on the association between mtDNA mutations and NPC [24]–[26]. For instance, a 4981-bp deletion has been detected in NPC tumors [24]; mtDNA variants T16362C, T16519C and mtDNA microsatellite instability at D310 (a poly-C stretch between mtDNA nucleotide position 303 and 315) are thought to be the risk factors for familial NPC [26], albeit these variants are scored as hypervariable sites in mtDNA control region [27]. However, the association between mtDNA haplogroups and NPC has been rarely reported. We have been studying on genetic variations on NPC susceptibility and previously reported the association of certain human leukocyte antigen (HLA)-A and -B alleles and haplotypes with NPC risk in Chaoshan population [28]. In this study, we investigated the possible association between mtDNA haplogroups and NPC in Chaoshanese based on the speculation that certain mtDNA haplogroup might confer susceptibility to NPC in high-risk areas. Our findings might give a clue to the development of NPC from the perspective of matrilineal genetic background.

## Materials and Methods

### Ethics Statement

The study was approved by the institutional review board of Shantou University Medical College (SUMC) in compliance with the ethical requirement of the Operational Guidelines for Ethics Committees That Review Biomedical Research (issued by the Ministry of Health of China in 2007) as well as the tenets of the Declaration of Helsinki. We interviewed each participant to obtain questionnaire-based written informed consents, which included permission to take peripheral blood samples. For participants under the age of 18 (statutory age in China), written informed consents were sought from the next of kin, caretakers, or guardians on the behalf of these participants. All the consents were kept securely and confidentially, and the information and the blood samples were used only for research purpose.

### Study Subjects

Peripheral blood samples were collected from 402 unrelated Chaoshanese, which comprised 201 patients with NPC and 201 matched normal controls (NC). All subjects were restricted to those who were born and whose families had resided in the Chaoshan region for more than two generations, but no restriction was strictly set for sex and age as previously described [28]–[30]. Patients were identified and pathologically confirmed at their initial visit between January 2001 to April 2004 for a nasopharyngeal examination in the Tumor Hospital, SUMC, which is a highly regarded teaching hospital serving the general population of the Chaoshan region. The NC subjects had no family and personal history of cancers and were randomly selected from those who had their annual physical examinations at the Physical Examination Center of the First Affiliated Hospital, SUMC. The age of the study subjects ranged from 13 to 73 years (mean age: 48.41±11.28 years) in the NPC group, and 18 to 87 years (mean age: 46.39±13.51 years) in the NC group. The male:female ratio was about 3.7∶1 (158∶43) and 1.7∶1 (126∶75) in the NPC and NC groups, respectively. To identify Chaoshanese and proper normal controls, a structured questionnaire was completed by each participant at recruitment to document demographic information, ethnic background, and family history of cancers.

To verify the ethnicity and potential population substructure of the studied subjects, mtDNA data of Chaoshanese from other reported studies as well as of other reported Han Chinese populations were collected for comparison. The detailed population sources are presented in Table S1 of the Supporting Information.

### PCR Amplification and Sequencing of mtDNA Control Region

Genomic DNA was extracted from whole blood using the methods of Chelex-100 [31] or salting-out extraction [32]. The two hypervariable segments (HVS-I at the region 16024–16383 and HVS-II at the region 57–372, numbering according to the revised Cambridge reference sequence (rCRS) [33]) of the mtDNA control region were amplified and sequenced for all samples using an asymmetric PCR approach as described [34]. Briefly, PCR primers were mixed in a 10∶1 ratio and the primer at the lower concentration was used up during the PCR, leaving the excess PCR primer as the sequencing primer in the next step. In this approach, PCR product was used directly in the following sequencing reaction without prior purification. PCR amplification was conducted in a total reaction volume of 10 µL, consisting of 1 µL of 10× reaction buffer, 1 µL 25 mM MgCl_2_, 0.5 µL 2 mM of each dNTP, 0.15 U Taq polymerase, 1.35 µL (Chelex-100 method) or 5 ng (salting-out method) DNA template, and 1 µL 1 µmol/L of heavy (or light) strand primer and 1 µL 10 µmol/L of light (or heavy) strand primer, with the cycling parameters of 3 min at 94°C for denaturation, followed by 35 cycles of 30 s at 94°C, 30 s at 58°C, 90 s at 72°C, and a final extension at 72°C for 7 min. PCR products were then subjected to direct sequencing using a Big-Dye Terminator v3.1 cycle sequencing kit and ABI 3100 automated DNA sequencer (Applied Biosystems, USA). All primers used for PCR amplification and sequencing are presented in Table S2.

### Detection of 9-bp COII/tRNA^Lys^ Intergenic Deletion

The mtDNA 9-bp deletion is caused by the loss of one copy of the 9-bp tandem repeat sequence (CCCCCTCTA) in the COII/tRNA^Lys^ intergenic region of human mtDNA and is usually used for determining haplogroup B status, along with the control region sequence motifs [35]. To detect the deletion, a PCR-polyacrylamide gel electrophoresis (PAGE) method [18], [36] was adopted using the primer pair L8215/H8297 (Table S2). This set of primers amplified 112-bp and 121-bp fragments, with one and two copies of the 9-bp tandem repeat, respectively. Briefly, the targeted region was PCR amplified and the amplified 112-bp and 121-bp fragments were then separated by 15% PAGE to confirm the presence of the 9-bp deletion.

### Genotyping of Other mtDNA Polymorphisms

For samples which could not be unambiguously classified based on the mtDNA control region sequence variations, either a PCR-restriction fragment length polymorphism (PCR-RFLP) analysis for haplogroup-specific coding region variants [18] was employed, or a coding region fragment (region 10171–10660 [33]) was sequenced, or both, to justify the haplogroup assignment. The sequencing procedure was the same as described above. The haplogroup-specific coding region variants analyzed by PCR-RFLP included A663G (recognized by +663 *Hae*III) for haplogroup A, A4833G (recognized by +4831 *Hha*I) for haplogroup G, C5178A (recognized by −5176 *Alu*I) for haplogroup D, and/or T9824C (recognized by +9820 *Hinf*I) for haplogroup M7 [18].

### mtDNA Haplogroup Assignment and Statistical Analysis

All sequences were scored relative to the rCRS [33]. The variants in each mtDNA sequence were recorded and further checked by using the MitoTool (www.mitotool.org) [37]. The haplogroup motifs were identified according to the mtDNA phylogeny for East Asian [36] and the PhyloTree for the global human mtDNA (www.phylotree.org; mtDNA tree Build 15, 30 Sep 2012) [38]. Each sample was classified, as possible, into the smallest named haplogroup based on all available haplogroup motifs identified. If the haplogroup had further named subhaplogroups, an asterisk was attached to the haplogroup name that refers to the mtDNA under consideration to emphasize that the haplogroup status of the mtDNA cannot be specified further (relative to the classification tree) [18]. This rule was particularly applied to those unassigned mtDNAs belonging to macrohaplogroups M, N or R. The mtDNA sequences generated in this study were deposited in GenBank under the accession numbers KC619327-KC619527 and KC741197-KC741397.

The mtDNA variant and haplogroup frequencies were computed jointly (for variants) and/or separately (for both) for NPC and NC groups. Binary logistic regression analysis was carried out to assess the association of each mtDNA sequence variant or inferred haplogroup with the risk of NPC by comparing their frequency distributions between cases and controls, and the strength of the relative risk was expressed as odds ratio (OR) and the OR with corresponding 95% confidence interval (95% CI). Stratified analyses were further performed to examine the effect of the mtDNA haplogroup background on NPC within strata of age (individuals ≥ or <40 years old) and/or gender (male and female groups). To avoid possible interference of age and gender on the results, the analysis was also adjusted for these two factors, in which each sequence variant and haplogroup was separately introduced into the regression equation with age and/or gender as independent variable(s). Principal component (PC) analysis was conducted to assess the clustering of the Chaoshanese analyzed in this study with the reported Chaoshan and other Han Chinese populations (Table S1) based on the mtDNA haplogroup frequencies. All statistical tests were two-sided and statistical significance was established at *P*<0.05. Unless specified, SPSS 13.0 package (SPSS Inc, Chicago, IL, USA) was used for statistical analyses.

## Results

### Clustering Pattern of Chanshanese with Other Han Chinese Populations

**Figure 1:**
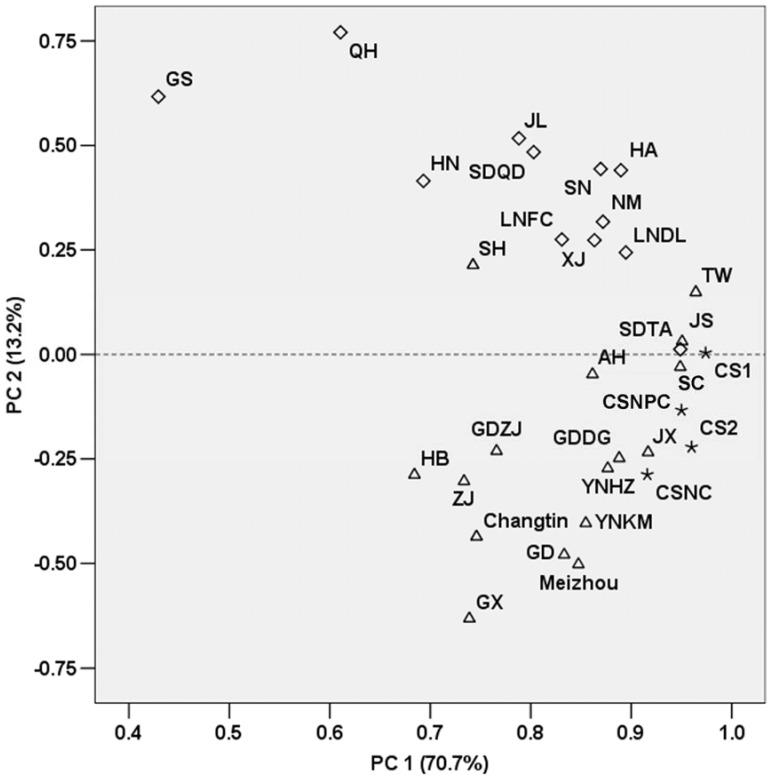
Principal component analysis of clustering between Chaoshanese and other Han Chinese populations. Nasopharyngeal carcinoma (NPC) patients and normal control (NC) subjects from the Chaoshan region in this study, together with reported Chaoshan populations [42.43] and other Han Chinese populations across China from published sources were included for analysis based on their mtDNA haplogroup frequencies. △ denotes southern Han populations, ◊ denotes northern Han populations, *denotes the Chaoshan populations. Each population is represented by the population ID as listed in Table S1.

To demonstrate that the NPC patients and control subjects analyzed in this study were generally homogenous and had no remarkable difference compared with those previously reported Chaoshan populations [39]–[41], we performed a PC analysis, based on mtDNA haplogroup frequencies, for the NPC and NC groups, together with the reported Chaoshanese [42], [43] and other Han Chinese populations (Table S1). As shown in Figure 1, the cumulative contribution of PC1 and PC2 accounts for 83.9% of the total variation, and the northern and southern Han populations each fall into their respective clusters, which are distinctly separated by PC2, with the northern Hans clustering together in the upper part of the plot and the southern Hans in the lower part. The NPC and NC populations are located closely within the southern Han population cluster. This pattern was well consistent with what we had previously reported on the genetic origin of the Chaoshanese by using both HLA [39] and short tandem repeat [40], [41] data. It is noticeable that our NPC and NC samples gathered with the two reported Chaoshan populations (CS1 [42] and CS2 [43]) within the southern Han population cluster (Figure 1), but these populations do not appear to be as closely as they were supposed to be. One possible reason for this pattern may lie in the sample composition since there is potential region difference within the Chaoshan area (Figure 1).

### mtDNA Control Region Sequence Variations and Their Association with NPC Risk

The mtDNA control region sequence variants and their distribution in NPC and NC groups are presented in Table S3. For the entire 676-bp fragment sequenced for HSV-I and HSV-II, we observed variations at 194 nucleotide positions (194/676, 28.70%) among 402 subjects of cases and controls combined. Because more than one variation occurred in some of the variable sites, totally 220 variants were identified. When comparing the variant frequency distribution between the NPC and NC groups, no difference was found in terms of the variable sites observed [NPC: 22.78% (154/676), NC: 23.82% (161/676); *P* = 0.652] and the variants detected [NPC: 25.30% (171/676), NC: 26.33% (178/676); *P* = 0.664]. However, as shown in Table 1, significant difference was observed for variant T204C (*P* = 0.005, OR = 0.34, 95% CI = 0.16–0.72), and the difference remained significant even after adjusting for age and gender (adjusted *P* = 0.003). Beside, the other two variants (G207A, and 249delA) also showed significant difference in frequency distribution between the two groups but only after age and gender adjustment. Of these three variants, 249delA (*P* = 0.033, OR = 1.68, 95% CI = 1.04–2.70) had higher frequencies in NPC patients (possibly reflects its higher presence in haplogroup R9c and its subhaplogroups in NPC population) than in healthy controls, but the frequency distribution of T204C (*P* = 0.003, OR = 0.31, 95% CI = 0.14–0.66) and G207A (*P* = 0.044, OR = 0.33, 95% CI = 0.11–0.97) showed the opposite tendency. Note that the significance of G207A was at a marginal level (Table 1) and such a comparison should be received with caution, as these variants were grouped as hypervariable sites in human mtDNA [27].

**Table 1:** mtDNA sequence variants in significant association with NPC risk in 201 NPC cases and 201 NC subjects from Chaoshan population.

Variant	NPC cases, *n* (%)	NC subjects, *n* (%)	*P*-value (adjusted^a^)	OR (95% CI)^a^
T204C	10 (4.98)	27 (13.43)	**0.005** (**0.003**)	0.31 (0.14–0.66)
G207A	5 (2.49)	12 (5.97)	0.092 (**0.044**)	0.33 (0.11–0.97)
249delA	56 (27.86)	41 (20.40)	0.081 (**0.033**)	1.68 (1.04–2.70)

Abbreviations: NPC, nasopharyngeal carcinoma; NC, normal control.

The value of P<0.05 is shown in bold.

a Adjusted for age and gender.

### mtDNA Haplogroup Distribution and Their Association with NPC Risk

All the subjects could be classified into known East Asian haplogroups [18], [36] (Table S4), as noted in the mtDNA tree of the PhyloTree (www.phylotree.org; mtDNA tree Build 15, 30 Sep 2012) [38]. Haplogroups that were prevalent in northern (haplogroups A, G, D, C, Z, M8a and Y) and southern (haplogroups B, F, M7b, R9b, and N9a) Chinese [18], [44], [45] were both observed, but there was no statistical difference (*P* = 0.118) concerning their overall frequency distributions between the NPC and NC groups.

As shown in Table 2, haplogroup R9 and its subhaplogroups R9c, F, F1, F1a'c, and F2 all presented significant differences between the NPC patients and the controls even after adjusting for age and gender. Haplogroup F1a joined in the significant difference when age and gender adjustment was applied. The NPC population significantly differed from the control population by having a higher frequency of haplogroup R9 (*P* = 0.021 with adjusted *P* = 0.011, OR = 1.91, 95% CI = 1.16–3.16), in particular of its main subhaplogroup F (*P* = 0.020 with adjusted *P* = 0.012, OR = 2.00, 95% CI = 1.17–3.41), indicating the association of these haplogroups with an increased risk for NPC. Distribution of the other haplogroups had no statistical difference between the case and the control groups (Table 2). Note that the significant values for haplogroup association did not maintain when the comparison was made between the NPC population and each of the two reported Chaoshan samples from the general populations [42], [43], but the trend for a higher frequency of R9 and its subhaplogroups in NPC remained unchanged (data not shown). The lack of consistence between different comparisons was possibly caused by the relatively small sample size of the reported Chaoshan populations [42], [43] and/or potential regional difference among Chaoshan populations. However when we combined the NC sample with the two reported Chaoshan samples from the general populations [42], [43] as a control population and compared with the NPC population, we observed a similar pattern of haplogroup association with NPC (Table 2).

**Table 2:** Frequencies of mtDNA haplogroups in NPC cases and NC subjects from Chaoshan population.

Haplogroup^a^	NPC cases (%)	NC subjects (%)	NPC versus NC		Chaoshan sample (%)	NPC versus CC	
			*P*-value (adjusted^b^)	OR (95% CI)^b^		*P*-value	OR (95% CI)
M	101(50.25)	103 (51.24)	0.842 (0.728)	0.93 (0.63–1.39)	206 (51.63)	0.750	0.95 (0.67–1.33)
D	44 (21.89)	31 (15.42)	0.097 (0.162)	1.45 (0.86–2.42)	79 (19.80)	0.549	1.14 (0.75–1.72)
D4	18 (8.96)	16 (7.96)	0.720 (0.803)	1.10 (0.54–2.24)	45 (11.28)	0.286	0.73 (0.41–1.31)
D4a	12 (5.97)	11 (5.47)	0.830 (0.914)	1.05 (0.45–2.47)	26 (6.52)	0.796	0.91 (0.45–1.85)
D5	20 (9.95)	10 (4.98)	0.062 (0.100)	1.95 (0.88–4.32)	26 (6.52)	0.138	1.59 (0.86–2.92)
D5a	9 (4.48)	5 (2.49)	0.283 (0.322)	1.77 (0.57–5.45)	7 (1.75)	0.059	2.63 (0.96–7.16)
M7	27 (13.43)	26 (12.94)	0.883 (0.935)	1.03 (0.57–1.84)	50 (12.53)	0.755	1.08 (0.66–1.79)
M7b	10 (4.98)	15 (7.46)	0.305 (0.365)	0.68 (0.29–1.57)	31 (7.77)	0.204	0.62 (0.29–1.30)
M7c	14 (6.97)	11 (5.47)	0.536 (0.761)	1.14 (0.50–2.60)	17 (4.26)	0.162	0.17 (0.81–3.49)
M8	11 (5.47)	15 (7.46)	0.419 (0.554)	0.78 (0.34–1.77)	30 (7.52)	0.350	0.71 (0.35–1.45)
CZ	7 (3.48)	12 (5.97)	0.245 (0.419)	0.67 (0.25–1.77)	21 (5.26)	0.332	0.65 (0.27–1.56)
C	6 (2.99)	5 (2.49)	0.760 (0.553)	1.45 (0.42–4.99)	11 (2.76)	0.874	1.09 (0.40–2.98)
M9	2 (1.00)	5 (2.49)	0.269 (0.350)	0.45 (0.08–2.41)	5 (1.25)	0.782	0.79 (0.15–4.2)
M10	4 (1.99)	6 (2.99)	0.525 (0.517)	0.65 (0.18–2.40)	11 (2.76)	0.572	0.72 (0.23–2.28)
M12'G	7 (3.48)	14 (6.97)	0.124 (0.082)	0.43 (0.17–1.11)	21 (5.26)	0.332	0.65 (0.27–1.56)
G	6 (2.99)	11 (5.47)	0.222 (0.159)	0.48 (0.17–1.34)	18 (4.51)	0.371	0.65 (0.25–1.67)
N	100 (49.75)	98 (48.76)	0.842 (0.728)	1.07 (0.72–1.60)	193 (48.37)	0.750	1.06 (0.75–1.48)
R9	52 (25.87)	33 (16.42)	**0.021** (**0.011**)	1.91 (1.16–3.16)	75 (18.80)	**0.046**	1.51 (1.01–2.26)
R9b	6 (2.99)	3 (1.49)	0.321 (0.235)	2.37 (0.57–9.89)	8 (2.01)	0.456	1.50 (0.52–4.40)
R9c	46 (22.89)	27 (13.43)	**0.015** (**0.009**)	2.05 (1.20–3.50)	64 (16.04)	**0.042**	1.55 (1.02–2.37)
F	45 (22.39)	27 (13.43)	**0.020** (**0.012**)	2.00 (1.17–3.41)	63 (15.79)	**0.048**	1.54 (1.00–2.36)
F1	26 (12.94)	13 (6.47)	**0.031** (**0.015**)	2.43 (1.18–5.00)	40 (10.03)	0.283	1.33 (0.79–2.26)
F1a'c	25 (12.44)	12 (5.97)	**0.028** (**0.012**)	2.60 (1.24–5.45)	32 (8.02)	0.084	1.63 (0.94–2.83)
F1a	23 (11.44)	12 (5.97)	0.056 (**0.027**)	2.33 (1.10–4.93)	30 (7.52)	0.112	1.59 (0.90–2.82)
F2	12 (5.97)	1 (0.50)	**0.015** (**0.012**)	14.29 (1.81–112.79)	7 (1.75)	**0.009**	3.56 (1.38–9.18)
F2b	6 (2.99)	0 (0.00)	0.999 (0.999)	UD (0.00-UD)	0 (0.00)	0.999	UD (0.00-UD)
B4'5	34 (16.92)	37 (18.41)	0.695 (0.618)	0.88 (0.52–1.48)	70 (17.54)	0.848	0.96 (0.61–1.50)
B4	28 (13.93)	27 (13.43)	0.885 (0.954)	1.02 (0.57–1.81)	55 (13.78)	0.961	1.01 (0.62–1.65)
B4a	10 (4.98)	6 (2.99)	0.313 (0.397)	1.57 (0.55–4.46)	18 (4.51)	0.799	1.11 (0.50–2.45)
B4b	8 (3.98)	6 (2.99)	0.588 (0.652)	1.29 (0.43–3.83)	11 (2.76)	0.422	1.46 (0.58–3.69)
B4c	8 (3.98)	8 (3.98)	1.000 (0.927)	1.05 (0.38–2.90)	14 (3.51)	0.772	1.14 (0.47–2.76)
B5	6 (2.99)	10 (4.98)	0.313 (0.277)	0.56 (0.20–1.60)	15 (3.76)	0.627	0.79 (0.30–2.06)
R11'B6	1 (0.50)	6 (2.99)	0.094 (0.088)	0.16 (0.02–1.32)	7 (1.75)	0.235	0.28 (0.03–2.29)
A	6 (2.99)	15 (7.46)	0.051 (0.068)	0.40 (0.15–1.07)	20 (5.01)	0.255	0.58 (0.23–1.48)
N9	5 (2.49)	4 (1.99)	0.737 (0.896)	1.09 (0.29–4.19)	15 (3.76)	0.416	0.65 (0.23–1.82)

Abbreviations: NPC, nasopharyngeal carcinoma; NC, normal control; CC, Chaoshan sample, which included NC in this study and the two reported Chaoshan populations [42], [43]; UD, undefined.

The value of *P*<0.05 is shown in bold.

a Haplogroups were nested, within the respective NPC or NC group, according to their phylogenetic positions in the global human mtDNA PhyloTree (www.phylotree.org; mtDNA tree Build 15, 30 Sep 2012) [38]. For example, M contains D and M* and their sub-haplogroups, and so does N which contains R* and N* and their sub-haplogrous.

b Adjusted for age and gender.

We further performed stratification analyses by dividing the subjects, within the respective NPC cases and controls, into gender (male and female), age (≥ and <40 years old), and age and gender (male with age ≥ or <40 years and female with age ≥ or <40 years old) subgroups. Interestingly, haplogroup R9 and its subhaplogroups R9c, F, F1, F1a'c, and F1a still maintained significant differences between the case and the control groups in the subgroups of the male and those with an age ≥40 years (Table 3), with a higher haplogroup frequency in the NPC cases as observed in the non-stratification analysis (Table 2). Although haplogroup R9 lost its significance (*P* = 0.091) and the significance of haplogroup F was abased to the marginal level (*P* = 0.059) in the subgroup of male subjects with age ≥40 years, all other subhaplogroups (R9c, F1, F1a'c, and F1a) maintained their significant association with NPC risk. More importantly, the association with NPC risk became obviously stronger when we branched down along the R9-F1-F1a'c-F1a limb, for which an over 4-fold (male subgroup), 3-fold (≥40 years subgroup), and about 5-fold (male with ≥40 years) increased risk for NPC were observed (Table 3), suggesting that the effect of haplogroup R9 on NPC might be attributable to its subhaplogroup F1. No any association with NPC was observed in the subgroups of the female, those who with an age <40 years, and the female with <40 years (Table 3). However, the sample size of female individuals in NPC was too small to make a firm conclusion. It should be noted that the significant association of F2 with NPC risk was lost in the stratification analyses. This might be due to sample size changes: there was only one F2 sample (male at age 45 years old) in the control group and 12 in the NPC group, and the numbers of F2 individual in the NPC group dropped to 8, 6, and 5, respectively, when the analysis was restricted to male individuals, to subjects with an age ≥40 years, or to male with age ≥40 years (data not shown). The effect of haplogroup F2 on NPC needs to be confirmed in future study with a larger sample size.

**Table 3:** Association of mtDNA haplogroups with NPC risk stratified by gender and age.

		Haplogroup carrying (*n*), +/−			
Stratification	Haplogroup	NPC cases	NC subjects	*P*-value	OR (95% CI)
*Gender* ^a^					
Male	R9	39/119	17/109	**0.019**	2.13 (1.13–3.98)
	R9c	35/123	14/112	**0.015**	2.30 (1.18–4.50)
	F	34/124	14/112	**0.020**	2.22 (1.13–4.36)
	F1	20/138	4/122	**0.007**	4.54 (1.51–13.70)
	F1a'c	19/139	3/123	**0.006**	5.74 (1.65–19.91)
	F1a	18/140	3/123	**0.008**	5.42 (1.55–18.91)
Female	R9	13/30	16/59	0.201	1.78 (0.74–4.32)
	R9c	11/32	13/62	0.140	2.05 (0.79–5.33)
	F	11/32	13/62	0.140	2.05 (0.79–5.32)
	F1	6/37	9/66	0.551	1.42 (0.45–4.49)
	F1a'c	6/37	9/66	0.551	1.42 (0.45–4.49)
	F1a	5/38	9/66	0.830	1.14 (0.34–3.80)
*Age (years)* ^b^					
≥40	R9	39/122	22/119	**0.047**	1.77 (0.99–3.18)
	R9c	35/126	17/124	**0.027**	2.04 (1.09–3.85)
	F	34/127	17/124	**0.036**	1.97 (1.05–3.72)
	F1	21/140	7/134	**0.015**	3.04 (1.24–7.45)
	F1a'c	20/141	6/135	**0.011**	3.43 (1.32–8.90)
	F1a	18/143	6/135	**0.025**	3.01 (1.15–7.88)
<40	R9	13/27	11/49	0.066	2.59 (0.94–7.15)
	R9c	11/29	10/50	0.107	2.39 (0.83–6.91)
	F	11/29	10/50	0.107	2.39 (0.83–6.91)
	F1	5/35	6/54	0.611	1.42 (0.37–5.47)
	F1a'c	5/35	6/54	0.611	1.42 (0.37–5.47)
	F1a	5/35	6/54	0.611	1.42 (0.37–5.47)
*Gender&Age*					
Male ≥40	R9	30/100	15/90	0.091	1.80 (0.91–3.56)
	R9c	28/102	12/93	**0.043**	2.13 (1.02–4.43)
	F	27/103	12/93	0.059	2.03 (0.97–4.24)
	F1	16/114	3/102	**0.015**	4.77 (1.35–16.85)
	F1a'c	15/115	2/103	**0.013**	6.72 (1.50–30.08)
	F1a	14/116	2/103	**0.017**	6.22 (1.38–28.00)
Male <40	R9	9/19	2/19	0.076	4.50 (0.86–23.64)
	R9c	7/21	2/19	0.181	3.17 (0.59–17.15)
	F	7/21	2/19	0.181	3.17 (0.59–17.15)
	F1	4/24	1/20	0.299	3.33 (0.34–32.27)
	F1a'c	4/24	1/20	0.299	3.33 (0.34–32.27)
	F1a	4/24	1/20	0.299	3.33 (0.34–32.27)
Female ≥40	R9	9/22	7/29	0.361	1.70 (0.55–5.26)
	R9c	7/24	5/31	0.359	1.81 (0.51–6.41)
	F	7/24	5/31	0.359	1.81 (0.51–6.41)
	F1	5/26	4/32	0.550	1.54 (0.37–6.32)
	F1a'c	5/26	4/32	0.550	1.54 (0.37–6.32)
	F1a	4/27	4/32	0.822	1.19 (0.27–5.19)
Female <40	R9	4/8	9/30	0.478	1.67 (0.41–6.84)
	R9c	4/8	8/31	0.365	1.94 (0.46–8.10)
	F	4/8	8/31	0.365	1.94 (0.46–8.10)
	F1	1/11	5/34	0.676	0.62 (0.07–5.88)
	F1a'c	1/11	5/34	0.676	0.62 (0.07–5.88)
	F1a	1/11	5/34	0.676	0.62 (0.07–5.88)

Abbreviations: NPC, nasopharyngeal carcinoma; NC, normal control.

The value of P<0.05 is shown in bold.

The analysis was adjusted for ^a^age or ^b^gender.

## Discussion

This study sought to investigate whether there was any link between mtDNA background (sequence variation and haplogroup distribution) and the risk of NPC, and came to an important identification of haplogroup R9, and in particular of its main sub-haplogroup F1, as a high-risk haplogroup for NPC in Chaoshan population. Haplogroups R9 and F1 had a significantly increased frequency in the NPC cases relative to the controls and conferred about 2 times or more of the NPC susceptible effect (Table 2). Interestingly, the association of the R9-F1 matrilineal background with NPC differed by age and gender, with significantly increased risk in males and/or individuals ≥40 years of age, but not in females and/or individuals <40 years of age (Table 3). The results agreed well with the epidemiological observation that NPC incidence, among those high-risk populations, increases with age and peaks at 45–54 years of age, and the male:female ratio for the disease incidence is about 2-3∶1 [46], [47]. It has been recognized that mtDNA mutations affect sex-specific patterns of certain biological features because maternal transmission of mtDNA acts as a sex-specific selective sieve, which enables accumulation of male-harming mutations in mtDNA when these same mutations are neutral, beneficial, or only slightly deleterious in their effects on females [48], [49]. Moreover, haplogroup R9, and especially F1, might be a specific risk factor for NPC, because a different factor, haplogroup D, is identified to be responsible for genetic susceptibility to esophageal carcinoma, another highly prevalent cancer in the same population studied [21], [23]. This indicates that different haplogroups may confer susceptibility to different cancers, although the exact mechanism remains to be explored. To the best of our knowledge, this is the first report showing a positive association between mtDNA haplogroups and NPC risk. Although our finding needs to be confirmed by independent studies, one might speculate that the higher rate of NPC among the Chaoshanese may be explained, at least in part, by a particular mtDNA background that has different frequency distributions between NPC cases and control subjects.

Haplogroup F comprises four subhaplogroups (F1-F4) in eastern Asia [36], [38]. Although the biological function of haplogroup F is unclear, a number of studies have linked it to certain human features and/or diseases, with either protective or harmful effects. For instance, haplogroup F is found to increase the risk for type 2 diabetes [50], [51] and this effect lies mainly with F1 [51]; in Han Chinese patients with Leber hereditary optic neuropathy (LHON), haplogroup F acts as a protective factor against the disease [52]–[54]; and a significant association of haplogroup F (especially subhaplogroup F3) with longevity is observed in the female Chuang population from Bama, Guangxi Province of China [55]. Haplogroup F has a distinctive distribution pattern in China: it is one of the dominant southern Han Chinese haplogroups but is low in frequency in northern Han Chinese [18], [45]. This distribution pattern indicates a different genetic background between southern and northern Chinese, and might point out one of the reasons that NPC is so largely common in south China but rare in north China [4]. Recent studies have provided evidence that mtDNA haplogroups affect cellular oxidative phosphorylation [56], in which haplogroup F functions in down-regulating oxidative phosphorylation but up-regulating glycolysis in type-2-diabetes cells [57]. Will haplogroup F exert its effect on NPC also by up-regulating glycolysis since cancer cells always adopt anaerobic metabolism as a mean of survival [12]? The exact role of haplogroup F in NPC awaits further investigation.

We also identified three mtDNA sequence variants that were significantly associated with the NPC risk, among which 249delA increased the risk, whereas T204C and G207A decreased the risk (Table 1). To evaluate these three variants, we compared our results with those of other allied studies [24]–[26], particularly with the one that seeks NPC-associated sequence variants mainly within the D-loop region [26] as we did here. In the reported study, the variants T16362C, T16519C, and D310 are found to be associated with an increased risk for NPC in pedigree members from the NPC-bearing families [26]. As shown in Table 1, the NPC-associated variants identified in these two studies are not the same, though both studies focus their investigations in the D-loop region. The variant T16519C was not reported in our study because it is beyond the region we sequenced. The variant D310 has been recognized as a mutational hot spot in several primary tumors [58]–[60] but no significant difference between our cases and control samples (*P* = 1.000) was observed. One possible reason that the sample size difference may cause this discrepancy could not be excluded. Variant T16362C is worth to be specially mentioned. As reported, this variant is exclusively associated with increased risk of familial, but not sporadic, NPC [26]. The result could explain our observation that no NPC association was found for this variant in our study, since our NPC cases were of sporadic type. It needs, however, to be emphasized that such an association analysis should be interpreted with extreme caution as these mtDNA variants always have a high recurrent mutation rate [27], and the association might be the result of complex interplay between a series of variants that belong to certain specific haplogroup. A point has actually been made that disrupting the mtDNA haplotype to simply count the occurrence of its variations, particularly for these hypervariable sites with a high recurrent rate, may lead to exaggeration of the role of these variants and neglect of the effect of the mtDNA haplotype/haplogroup [61].

There are several limitations of the current study. First, we did not conduct Bonferroni adjustment systematically in consideration of that correction were likely to miss significant differences because the adjustment is very conservative and the result always comes at the cost of increasing the probability of Type I error [62]. Second, we analyzed mtDNA haplogroups based mainly on polymorphisms in the control region. This cannot exclude a possibility that mutations in the coding region might be associated with NPC. Third, the sample size was limited, particularly for the female samples in the NPC group. We are currently collecting more samples to further assess our observation in future study. We also expect our findings could be confirmed by independent studies with a larger sample size.

In summary, we analyzed the influence of mtDNA background on NPC in the Chaoshan population that has a high prevalence of this cancer. Haplogroup R9, especially it sub-haplogroup F1, is likely to be a risk factor of NPC in Chaoshanese, especially in males and individuals ≥40 years of age. The pathogenesis of NPC is affected by a complex interaction of nuclear genes, viruses and environmental factors, these factors might interact with mtDNA in NPC patients and increase the risk of the disease. Further study combined with these factors might give a clue to understand the role of mtDNA alterations in NPC.

## Supporting Information

Table S1 Detailed sources of the Han Chinese populations reanalyzed in this study.
(DOC)

Table S2 Primers used for PCR amplification, sequencing, and PCR-RFLP analysis in this study.
(DOC)

Table S3 mtDNA sequence variations and their distribution in Chanshanese with and without NPC.
(XLS)

Table S4 mtDNA sequence variation and haplogroup classification of 402 Chanshanese with and without NPC.
(XLS)
