# Supplementary material for: Mitochondrial DNA Haplogroup Confers Genetic Susceptibility to Nasopharyngeal Carcinoma in Chaoshanese from Guangdong, China
Source: PLoS One. 2014 Jan 31;9(1):e87795. doi: 10.1371/journal.pone.0087795 (PMC3909237; doi:10.1371/journal.pone.0087795)
Supplement: Table S1 — Detailed sources of the Han Chinese populations reanalyzed in this study. (DOC) [file pone.0087795.s001.doc]

**Table S1**

Detailed sources of the Han Chinese populations involved in this study.

| Geography | Population ID | Locality | No. of samples | References |
| --- | --- | --- | --- | --- |
| Southern China | CSNC | Chaoshan, Guangdong | 201 | This study |
|  | CSNPC | Chaoshan, Guangdong | 201 | This study |
|  | CS1 | Chaoshan, Guangdong | 102 | [1] |
|  | CS2 | Chaoshan, Guangdong | 96 | [2] |
|  | Meizhou | Meizhou, Guangdong (Hakka) | 170 | [1] |
|  | Changtin | Changtin Fujian (Hakka) | 54 | [3] |
|  | AH | Anhui | 42 | [3] |
|  | JS | Jiangsu | 67 | [3] |
|  | SH | Shanghai | 56 | [3] |
|  | ZJ | Zhejiang | 61 | [3] |
|  | TW | Taiwan | 155 | [4] |
|  | HB | Hebei | 42 | [3] |
|  | HN | Hunan | 98 | [3,5] |
|  | JX | Jiangxi | 23 | [3] |
|  | SC | Sichuan | 70 | [3] |
|  | YNKM | Kunming, Yunnan | 43 | [6] |
|  | YNHZ | Huize, Yunnan | 58 | [3] |
|  | GX | Guangxi | 26 | [3] |
|  | GD | Guangdong | 69 | [7] |
|  | GDDG | Dongguan, Guangdong | 105 | [8] |
|  | GDZJ | Zhanjiang, Guangdong | 30 | [6] |
| Northern China | XJ | Xinjiang | 47 | [6] |
|  | GS | Gansu | 45 | [3] |
|  | QH | Qinghai | 44 | [3] |
|  | LNFC | Fengcheng, Liaoning | 51 | [6] |
|  | LNDL | Dalian, Liaoning | 51 | [3] |
|  | NM | Neimenggu | 45 | [6] |
|  | SN | Shaanxi | 138 | [3,5] |
|  | HA | Henan | 198 | [1] |
|  | SDQD | Qingdao, Shandong | 50 | [6] |
|  | SDTA | Taian, Shandong | 76 | [6] |

CSNPC and CSNC indicate the Chaoshan populations with and without NPC, respectively.

**Supplementary references**

1. Wang WZ, Wang CY, Cheng YT, Xu AL, Zhu CL, et al. (2010) Tracing the origins of Hakka and Chaoshanese by mitochondrial DNA analysis. Am J Phys Anthropol 141: 124-130.
2. Wang Q, Wang PF, Li SQ, Xiao XS, Jia XY, et al. (2010) Mitochondrial DNA haplogroup distribution in Chaoshanese with and without myopia. Mol Vis 16: 303-309.
3. Wen B, Li H, Lu D, Song X, Zhang F, et al. (2004) Genetic evidence supports demic diffusion of Han culture. Nature 431: 302-305.
4. Tsai LC, Lin CY, Lee JC, Chang JG, Linacre A, et al. (2001) Sequence polymorphism of mitochondrial D-loop DNA in the Taiwanese Han population. Forensic Sci Int 119: 239-247.
5. Oota H, Kitano T, Jin F, Yuasa I, Wang L, et al. (2002) Extreme mtDNA homogeneity in continental Asian populations. Am J Phys Anthropol 118: 146-153.
6. Yao YG, Kong QP, Bandelt HJ, Kivisild T, Zhang YP (2002) Phylogeographic differentiation of mitochondrial DNA in Han Chinese. Am J Hum Genet 70: 635-651.
7. Kivisild T, Tolk HV, Parik J, Wang Y, Papiha SS, et al. (2002) The emerging limbs and twigs of the East Asian mtDNA tree. Mol Biol Evol 19: 1737-1751.
8. Chen F, Wang SY, Zhang RZ, Hu YH, Gao GF, et al. (2008) Analysis of mitochondrial DNA polymorphisms in Guangdong Han Chinese. Forensic Sci Int Genet 2: 150-153.
