# Supplementary material for: Mitochondrial DNA Haplogroup Confers Genetic Susceptibility to Nasopharyngeal Carcinoma in Chaoshanese from Guangdong, China
Source: PLoS One. 2014 Jan 31;9(1):e87795. doi: 10.1371/journal.pone.0087795 (PMC3909237; doi:10.1371/journal.pone.0087795)
Supplement: Table S2 — Primers used for PCR amplification, sequencing, and PCR-RFLP analysis in this study. (DOC) [file pone.0087795.s002.doc]

**Table S2**

Primers used for PCR amplification, sequencing, and PCR-RFLP analysis in this study.

| Primer name | Sequence (5'→3') | Usage |
| --- | --- | --- |
| L15933^a^ | CAGTCTTGTAAACCGGAGATG | HVS-I amplification and sequencing |
| H16401^a^ | TGATTTCACGGAGGATGGTG | HVS-I amplification |
| H16488^a^ | AGGAACCAGATGTCGGATACAG | HVS-I amplification and sequencing |
| L16524^a^ | AAGCCTAAATAGCCCACACGTT | HVS-I amplification and sequencing |
| L48^a^ | CTCACGGGAGCTCTCCATGC | HVS-I amplification |
| H408^a^ | CTGTTAAAAGTGCATACCGCCA | HVS-I amplification and sequencing |
| L8215^b^ | ACAGTTTCATGCCCATCGTC | 9-bp deletion |
| H8297^b^ | ATGCTAAGTTAGCTTTACAG | 9-bp deletion |
| L10170^b^ | ACATAGAAAAATCCACCCCTTACG | Coding region (10171-10659) sequencing |
| H10660^b^ | TTCGCAGGCGGCAAAGACTA | Coding region (10171-10659) sequencing |
| L394^b^ | CACCAGCCTAACCAGATTTC | Haplogroup A (+663*Hae*III） |
| H902^b^ | GACTTGGGTTAATCGTGTGAC | Haplogroup A (+663*Hae*III） |
| L4887^b^ | TGACAAAAACTAGCCCCCATCT | Haplogroup D (-5176 *Alu*I (5178A)) |
| H5442^b^ | GCGATGAGTGTGGGGAGGAA | Haplogroup D (-5176 *Alu*I (5178A)) |
| L4499^b^ | TGGCCCAACCCGTCATCTAC | Haplogroup G (+4831*Hha*I (4833)) |
| H5099^b^ | GGAATGCGGTAGTAGTTAGG | Haplogroup G (+4831*Hha*I (4833)) |
| L9794^b^ | GACGGCATCTACGGCTCAACA | Haplogroup M_7_ (+9824 *Hinf*I (9824)) |
| H10164^b^ | GAAGCCGCACTCGTAAGG | Haplogroup M_7_ (+9824 *Hinf*I (9824)) |

^a^ Primers designed in this study using the Primer Premier 5.0 program.

^b^ Primers adopted from the reference [1].

**Supplementary reference**

1. Yao YG, Kong QP, Bandelt HJ, Kivisild T, Zhang YP (2002) Phylogeographic differentiation of mitochondrial DNA in Han Chinese. Am J Hum Genet 70: 635-651.
